# Surface Characterization of Lipid Biomimetic Systems

**DOI:** 10.3390/membranes11110821

**Published:** 2021-10-27

**Authors:** Anibal Disalvo, Maria A. Frias

**Affiliations:** Applied Biophysics and Food Research Center (Centro de Investigaciones en Biofísica Aplicada y Alimentos, CIBAAL), National University of Santiago del Estero and CONICET, Santiago del Estero 4206, Argentina; marafrias@hotmail.com

**Keywords:** lipid membranes, biomimetic systems, hydration, dipole potential, zeta potential

## Abstract

Zeta potential and dipole potential measures are direct operational methodologies to determine the adsorption, insertion and penetration of ions, amphipathic and neutral compounds into the membranes of cells and model systems. From these results, the contribution of charged and dipole groups can be deduced. However, although each method may give apparent affinity or binding constants, care should be taken to interpret them in terms of physical meaning because they are not independent properties. On the base of a recent model in which the lipid bilayer is considered as composed by two interphase regions at each side of the hydrocarbon core, this review describes how dipole potential and zeta potential are correlated due to water reorganization. From this analysis, considering that in a cell the interphase region the membrane extends to the cell interior or overlaps with the interphase region of another supramolecular structure, the correlation of dipole and electrostatic forces can be taken as responsible of the propagation of perturbations between membrane and cytoplasm and vice versa. Thus, this picture gives the membrane a responsive character in addition to that of a selective permeability barrier when integrated to a complex system.

## 1. Introduction

When dry lipids such as phosphatidylcholines are dispersed in water to form bilayers enclosing an aqueous media or spread on the air–water surface to form monolayers, they interact strongly with water. These systems have been used extensively as experimental model system to study the behavior of the lipid membranes in cells. Using different controlled concentrations, studies in vitro try to mimic the behavior in real membranes of living cells. This is why they in general are defined as biomimetic systems.

**Figure 1:**
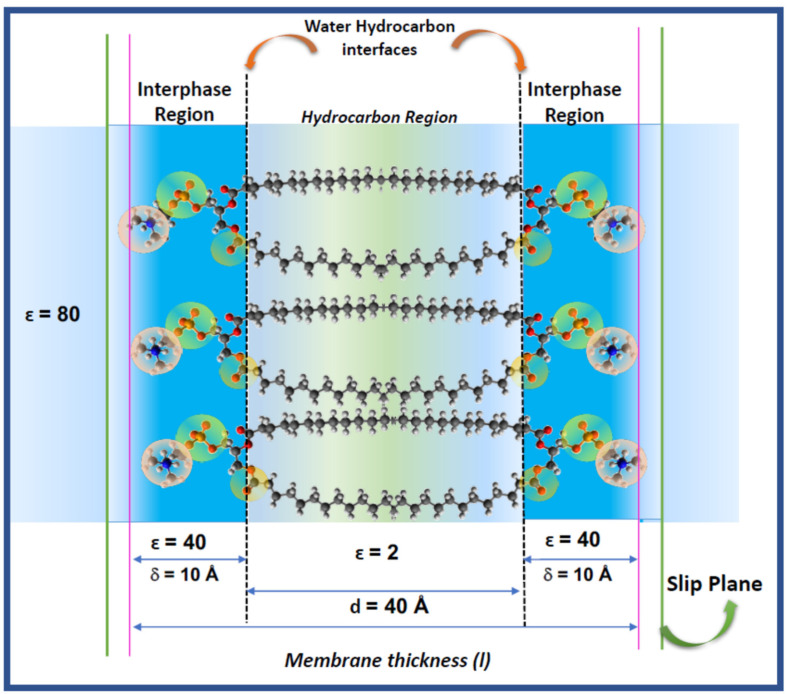
The bidimensional solution model for lipid membrane. The interphase region consists of a solution of polar head groups and its hydration shell (circles) immersed in water (blue region) of different properties than the bulk (light blue). In this region, constituent dipoles such as CO and PO groups are oriented in different directions. Details of water dipoles are not included for simplicity. The ascribed value for the dielectric permittivity at the different regions is denoted. The total thickness is obtained as l = 2δ + d, where δ is the thickness of the interphase and d the thickness of the hydrocarbon region. The region denoted by δ is that in which the electrical double layer (EDL) is formed. For details see text [8,9,10,11].

The hydration of the lipids is determinant for the thermodynamic stability and structural dynamics and are a clue to understand the response of the membrane to environmental changes and biologically relevant bio-effectors (hydric and oxidative stress, hydrolytic enzymes, signal peptides, antioxidants). A recent model of lipid membranes includes explicitly the lipid–water interaction considering the membrane as composed by a bidimensional solution of hydrated polar head groups of phosphatidylcholines, named the interphase [1,2,3,4,5,6,7] (Figure 1). 

This model can be sustained in terms of the formalism of Thermodynamics of Irreversible Processes, in which the membrane is considered as an open system with respect to water exchange. This exchange determines the mechanical changes due to osmotic expansion and contraction [2].

The thermodynamic analysis of the membrane has been done disregarding the electrical properties that can derive from the presence of charged groups (PO and NH groups), the orientation of dipolar groups (PO and CO), the orientation of multidipoles (P = N group) and the organization of water around them in that confined region. The molecular counterpart of this model indicates that water in the interphase is distributed around those membrane groups and the first -CH_2_ residues of the acyl chains. This ensures that the limits schematically represented in Figure 1 are not sharped.

Thus, if properties of the interphase region of lipid and cell membranes lays on the specific properties of water and its role in its functional response, the electrical properties derived from the distribution of charges and dipoles in the polar region of Figure 1 should be considered explicitly.

The distribution of charged groups, dipoles from the lipid residues and water dipoles determines, approximately within the limits of the interphase in Figure 1, an electric double layer (EDL) that stablishes an electrical barrier for interacting compounds and is susceptible to conformational changes by the action of physical and chemical variables which would explain the responsive properties of the membrane to changes in its adjacencies. This is not a trivial matter, since models of membranes should be considered in the context of the cells as a complex system, i.e., the link between chemical processes in cytoplasm and membrane phenomena [3].

In this regard, water layers around the charged and dipole residues of the lipids can give place to an environment of particular dielectric and polarization properties that affect the drop in the surface electric potential between the plane of the membrane and the water solution. Although zeta (charge potential) and dipole potential measures have been used routinely in membrane biophysics to analyze different kinds of bioactive compounds, a consistent view in terms of the thermodynamic and structural properties of the interphase region, considering water as fundamental component, is lacking.

For this reason, the purpose of this article is to describe the organization of charges and dipoles that define the electrical surface potentials of a lipid membrane and its dynamics in the lipid interphase to give a more complete insight on the physical chemical properties of lipid membranes.

The inclusion of electrical forces at the membrane surface may contribute to a better understanding of the mechanisms of insertion of free amino acids, oligo peptides, oligosaccharides, antioxidants and enzymes in relation to different levels of hydration of the membranes.

In the first part of the review, the different electrical potentials conveying in the interphase region will be described as operational tools to determine membrane–compounds interactions.

In the last part, an insight on the correlation between the different potentials will be discussed in order to explore cooperative and synergistic phenomena which are the essence of the membrane responsiveness

## 2. Definitions of Surface Potentials

The electric potentials associated to a surface can be divided in two contributions: the charge potential due to net charged residues (Volta potential, ψs) and the contribution of the orientation of dipoles (dipole potential, ψ_D_) *. All together determine the surface potential (Galvani potential, φ)

φ = ψs + ψ_D_ (1)

* This potential is usually denoted by the χ symbol in electrochemistry books.

The Galvani potential is the work achieved in bringing a unit charge from infinity in solution to the interior of a phase through the electric double layer composed by charges and dipoles.

The Volta potential is defined by the work in bringing the unit charge from infinity in solution to a point outside the phase such that the image forces are negligible and the charge does not penetrate the surface layer.

The dipole potential (ψ_D_) corresponds to the work in bringing the charge across the non-charge dependent potential, i.e., across the interface plane on which there is zero charges.

The Volta potential is the ordinary electric potential given by:

Ψ = 4πqδε(2)

where q is the charge, δ the separation of charges, ε the dielectric permittivity of the medium between the charges.

In turn, the dipole potential is given by:

ψ_D_ = 4πΝμ/ε (3)

Equation (3) is valid for N number of dipoles all of them with a dipole moment (μ) and (ε) the dielectric permittivity.

In terms of membrane structure, the Galvani potential would be representative of the work to bring the charge from the bulk solution to the membrane interior, i.e., the hydrocarbon region represented by the chains in Figure 1. This implies to overcome the interphase region δ of dielectric constant ε = 40. Therefore, the interphase can be considered qualitatively similar to the electrical double layer.

In turn, the Volta potential counts for the work to bring the charge up to the inner limit of that interphase (the water/hydrocarbon interface in Figure 1) without penetrating into the hydrocarbon phase.

The dipole potential is due to the work against the noncharged dependent potential, i.e., the dipoles.

The transcription of these definitions to a lipid membrane needs additional clarifications in regard to the model of Figure 1. The so-called phase in the Galvani and Volta definition implies a homogeneous isotropic media, which is not precisely the case for a lipid membrane. It is implicit in this definition that if the membrane is a phase, the transference into it can be resemble to a partition. However, this is questionable. That “phase” is d = 40 Å thick (two extended and opposed acyl chains of 20 Å each). Usually, the definition of partition in lipid membranes deduced from fluorescent probes, do not account for electrical contributions. Secondly, the membrane interior is not an isotropic and autonomous phase as the partition rule (Henry’s law) requires. On the contrary, water accompanies the solute solubilizing in the membrane core and contributes to its stabilization. This process involves changes in membrane density due to expansion (area changes) and mechanical (bending and distortions) properties [4,5].

In regard to the dipole potential, defined by Equation (2), the dipoles or multidipoles contributing to it are of different magnitudes which in turn varies according to the solid angle that they may form with the membrane plane taken as reference. This variation is dependent on the lipid membrane packing, i.e., surface pressure and osmotic state.

Finally, there is another point of ambiguity in both definitions in relation to the value of the dielectric permittivity in Equations (2) and (3), ascribed to the presence of water. These have been dispersed between 40 and 60, all of them much lower than that corresponding to bulk water (ε = 81). Thus, the influence of the lipid membrane residues and its state affect the water properties in the EDL.

Therefore, there are several structural features specific of the lipid membranes nor explicitly considered that results in simplifications and assumptions.

In electrical terms, considering its lipid nature described above, the bilayer can be described as a thin slab of non-conductive material with a capacitance given by:

(4) $$C\;=\;\varepsilon \;\times {\;\varepsilon }^{0}\;\frac{A}{d}$$

where (ε^0^) is the permittivity of free space, (d) the distance between the two planes in the membrane that constrain the hydrocarbon region of a dielectric constant (ε) equal to 2 and A is the area. The capacitance of a lipid membrane measured in a planar lipid bilayer set up (BLM) is around 1 μF/cm^2^, for a distance d = 50 Å, a value that does not fit to a pure capacitor [6]. This can be due to the fact that according to Figure 1, the bilayer is not a simple capacitor but the juxtaposition of the hydrocarbon slab with the bidimensional ionic solutions defined as the interphase regions at each side.

Thus, the membrane thickness can be taken as that including the two hydrocarbon chains of 20 Å each (d = 40 Å) and the aqueous interphase thickness δ, i.e. the EDL (two chains of 10 Å thick). Thus, the total membrane thickness is 60 Å [7].

In consequence, in order to define in a properly the electrical behavior of a lipid membrane, the origin of the surface potential in terms of the membrane components should be described on the base of the bidimensional solution model depicted in Figure 1.

## 3. Charge Potential and Zeta Potential

The surface potential (ψs) in a membrane is defined by the difference in electrostatic potential between the membrane surface and the bulk of the solution adjacent to the membrane. The membrane surface is the internal plane of the interphase in Figure 1, i.e., the dotted line along the glycerol/carbonyl region.

**Figure 2:**
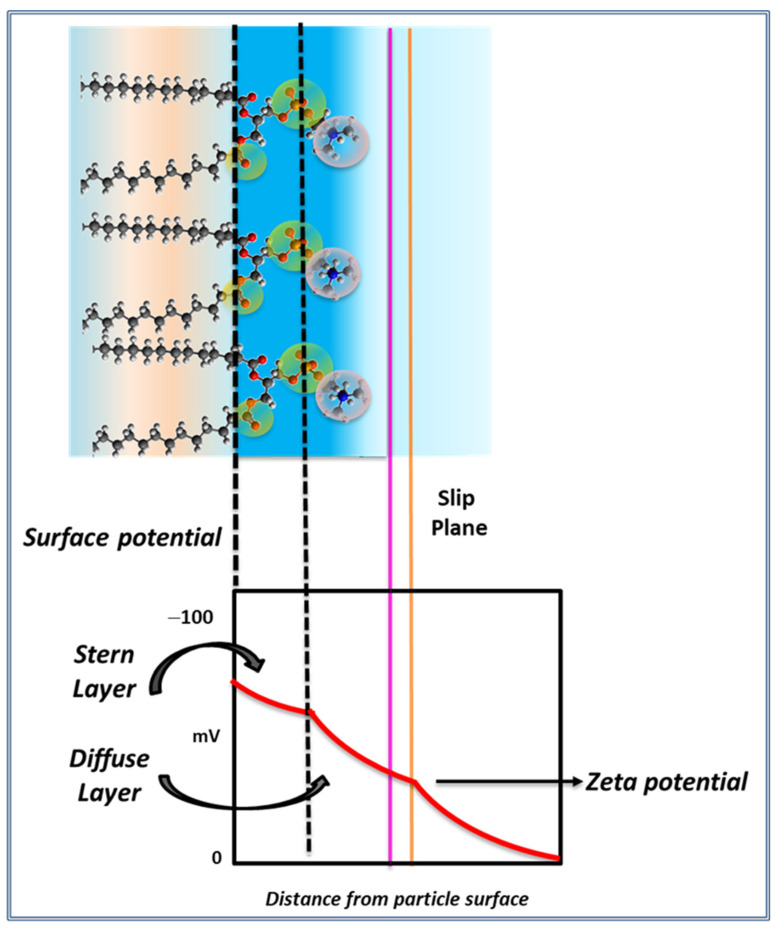
Electrical potential at the membrane surface.

The electric potential decreases with the distance from the surface of the particles as:

Ψ_s_= Ψ_d_ e^−Kδ^ (5)

where ψ = surface potential at a distance δ of the Stern layer, ψ_d_ = surface potential at the Stern layer, κ = Debye–Hückel parameter that depends on the ionic strength, δ = distance, the interphase thickness. This layer is called the diffuse or outer Helmholtz layer and can be modified by the ionic strength (Figure 2) In general, the modification of this layer by changes in the surrounding media can affect the electric mobility of the particle. This will be better described later.

The combination of these potentials determines the surface potential and both, at each side of the bilayer, results in the (Δψ) transmembrane potential [11].

However, this potential is experimentally inaccessible. For this reason, zeta potential is measured to determine the properties related to surface charges [12]. This merits some precisions.

The surface charge potential in lipid membranes is usually determined in liposome or vesicle dispersions by microelectrophoresis. The application of an electric field to such suspensions produces a rectilinear and uniform displacement of the particle to the electrode of opposite charge. The ratio between the rate of displacement to the applied field defines the electrophoretic mobility (*u*). This is an intrinsic property of the particle.

The measured electrophoretic mobility (*u*) is converted into zeta potential (ζ) through Henry’s equation:

*u* = 2εζ*F*(κa)/3η (6)

where ε is the dielectric constant of the dispersant, *F*(k*a*) is the Henry function and η is the viscosity [13,14]. This equation strictly applies for isolated particles of zeta potential less than around 25 mV.

The zeta potential or electrokinetic potential is calculated, mostly using the Helmholtz–Smoluchowski Equation (4) for *F*(k*a*) = 1.5 where k*a* is large and the double layer is thin in comparison with the particle radius.

(7) $$\zeta =\frac{\eta \mu }{\varepsilon \varepsilon_{0}}$$

where $\varepsilon ,\;\varepsilon_{0}$ and η correspond to the dielectric permittivity of the aqueous solution, the dielectric permittivity of vacuum and the viscosity of the suspension, respectively [14].

This equation applies to particles of diameter ≥1 μm in aqueous solution of high electrolyte concentration (≥10^−2^ M).

The zeta potential is defined as the potential drop between the surface of the membrane and the limit of the stagnant layer (the slip plane in Figure 2). Thus, hydrodynamic features are important in its determination such as, shape, size and viscosity. Therefore, there is a slight difference with the real charge potential [13].

That is why it is more precise to determine the electrophoretic mobility of the particle, especially when properties of the membrane have to be derived.

Performing electrokinetic measurements and interpreting their results in terms of ζ-potential should be independent of the technique used for its determination. However, the ζ-potential can be misinterpreted in terms of the conductivity of the stagnant layer, the shape of the particle and the application range of the theories. A thorough description of the main electrokinetic methods and the electrokinetic consistency tests has been extensively discussed elsewhere and we refer to them [14].

The origin of the zeta potential can be ascribed to the net charges of the lipid constituents immersed at the interphase such as, phosphates and amino groups and its combinations according to the lipid composition in phosphatidylcholines, phosphatidylethanolamines, phosphatidylglycerol, phosphatidic acids, among others (Figure 2). These charges on the external region of the membrane impose a distribution of counter ions that finally determines the actual surface charge potential. This distribution of charges is known as the electrical double layer (EDL) [15].

The EDL is described to be composed by two regions: the Stern layer (also named the inner Helmholtz layer) which is determined by the lipid charged groups (PO and NH) and its counterions, and another layer which depends on the temperature.

In terms of the EDL, the origin of the sign of the zeta potential is a matter of discussion. It is related with the microscopic arrangement of groups and water in the interphase depicted in Figure 1 and Figure 2. A proposal ascribes its sign and value to the orientation of the polar head groups (P-N multidipole in Figure 2) in the plane of the membrane. This orientation varies with temperature and ionic strength [16].

Phosphatidylcholines (PC) and phosphatidylethanolamines (PE) are the most abundant zwitterionic lipids in cell membranes and according to Makino et al. [16] their polar heads can reorient depending on the ionic strength. The zeta potential changes with the ionic strength can be interpreted via changes of the polar head group orientation, as well by compression of the diffuse part of electric double layer. At a low ionic strength, the choline groups are located below the phosphate group (negative zeta potential), whereas at a high ionic strength the situation is reversed.

Another interpretation considers that the zeta potential of vesicles containing anionic lipids can be ascribed to the specific adsorption of certain ions to the charged surface. Tatulian showed that the zeta potential varied according to the sequence ClO_4_^−^, > I^−^ > SCN^−^ > Br^−^ > NO_3_^−^, > Cl^−^ = SO_4_^2−^ denoting the importance of the polarizability of the anion [17]. Considering that Cl^−^ is present in biological media it would explain why liposomes and vesicles composed by neutral phospholipids such amphiphilic phosphatidylcholines bear a negative zeta potential. In Tatulian terms, chlorides would be adsorbed to the lipid interphase [17].

In a recent analysis, it is stated that the sign of the zeta potential cannot be predicted directly from the head group composition, since it strongly depends on the phase state and the ionic media in multilamellar and unilamellar lipid vesicles [18]. As in the phase transition water content changes from 7 water/lipid in the gel state to 22–24 in the liquid crystalline one, the influence of water cannot be discarded. In addition, as it will be shown in Section 6, dipole potential also varies in gel-LC transition, meaning a direct reorganization of water dipoles [19].

## 4. Methodologies

Zeta potential measurements are carried out using a technique called microelectrophoresis. For this measurement the sample is placed in a viewing chamber called an electrophoresis cell. Then, an electric field is applied. This causes the particles to move with a velocity that is proportional to their zeta potential in a direction that indicates whether their charge is positive or negative [18,20,21]. The method allows to measure multilamellar or unilamellar vesicles. The multilamellar liposomes are usually prepared by resuspending the lipid in water about the phase transition. Unilamellar vesicles are usually prepared from multilamellar vesicles by extrusion through membranes with different pore diameter. Lipid vesicles are observed and tracked using video optics or a high-quality stereo microscope. The advantage of this technique is that it is a measure on individual particles. Thus, usually the concentration is low to avoid interferences. A generous number of measures selecting different particles gives an average value of mobility and zeta potential. In addition, a detail inspection of the deviation of zeta potential allows to build a histogram of zeta potential distribution according to particle sizes [20].

Another set up for zeta potential determinations is based on dynamic light scattering. In this case, the system works according to the PALS (phase analysis light scattering) principle, and the data are automatically evaluated on the basis of the Smoluchowski equation (the particle size of ≈100 nm is much larger than the Debye length, ≈1 nm) [18].

Dynamic light scattering can also be applied for the determination of the particle sizes and their distributions. The size and distribution (percentage) of the particles (liposomes) evaluated from intensity of the dispersed light is a basic parameter to calculate zeta potential according to the Henry equation [14].

## 5. Zeta Potential Applications

### 5.1. Zeta Potential Measures

In principle, application of ζ-potential-based methodologies requires charged molecules in the system in order to measure surface charge alterations when molecules interact with liposomes [22]. However, this requirement is not exclusive since, several physical conditions and neutral chemical compounds can change the zeta potential as a result of changing membrane packing, phase state or water content at the level of the interphase (EDL) region, based on the properties inserted in its definition by Equation (7).

A direct determination of the binding of ions to lipid membranes, interactions of amino acids, and peptides, antioxidants, and proteins can be achieved simply measuring the relative change of zeta potential as a function of concentration. Protein adsorption can also be determined with or without enzymatic activity [12,23].

### 5.2. Ion Adsorption

**Figure 3:**
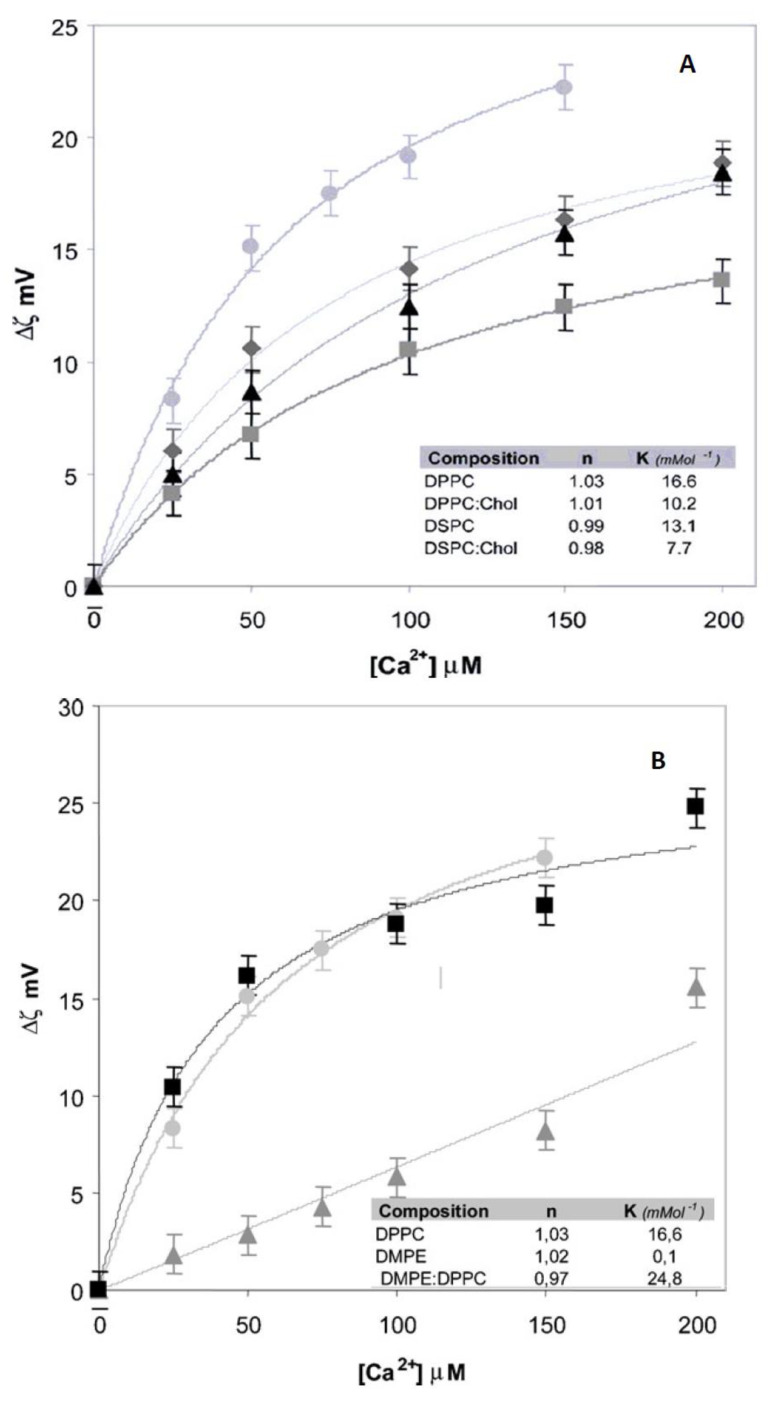
Changes of zeta potential induced by Ca^2+^. (**A**) Effect of acyl chain and cholesterol, DPPC (〇); DSPC (◆); DPPC-Cholesterol (Δ); DSPC-Cholesterol (☐). (**B**) Effect of polar head group. DPPC (☐); DMPC-DMPE (〇); DMPE (Δ) [24].

In Figure 3, it is observed that the addition of Ca^2+^ to liposomes of different composition produces significant changes in zeta potential which evolves to lower negative values in comparison to the liposomes in the absence of the ion. The adsorption is severely affected by the acyl chain nature and the presence of cholesterol (Figure 3A).

These data illustrate that lipids have different properties of adsorption although they are chemically neutral. The effect of Ca^2+^ can be explained as a direct interaction of the ion with the negative phosphate in the membrane, incorporating to the Stern layer. 

The difference observed between PC and PE denotes the importance of the hydration level in this process (Figure 3B). The first contains 22–25 water molecules per lipid and PE less than 7. Thus, hydration can be crucial to zeta potential and adsorption of cations.

In this regard, it is also interesting to denote that Ca^2+^ adsorption features vary significantly in the absence of CO groups. These groups act as hydration sites polarizing water and determine the dipole potential as will be described in next section. This charge adsorption is not independent of water and dipole arrangements.

In addition, zeta potential changes significantly when bilayers go from a less hydrated state (gel phase) to a high hydrated state (liquid crystalline) independent of the changes in sign [18]. This is a clear indication that zeta potential regardless the distribution of ions, changes with the water level.

### 5.3. Degree of Coverage, Affinity Constant and Cooperativity

The traditional approach to interpret zeta potential data in terms of membrane adsorption has been to measure ζ as a function of the additive concentration at fixed lipid content. The resulting curve is fitted by a Langmuir isotherm or a Langmuir–Hill curve [24,25,26] or alternatively, transformed to ζ versus square root of concentration for a linear fit.

**Figure 4:**
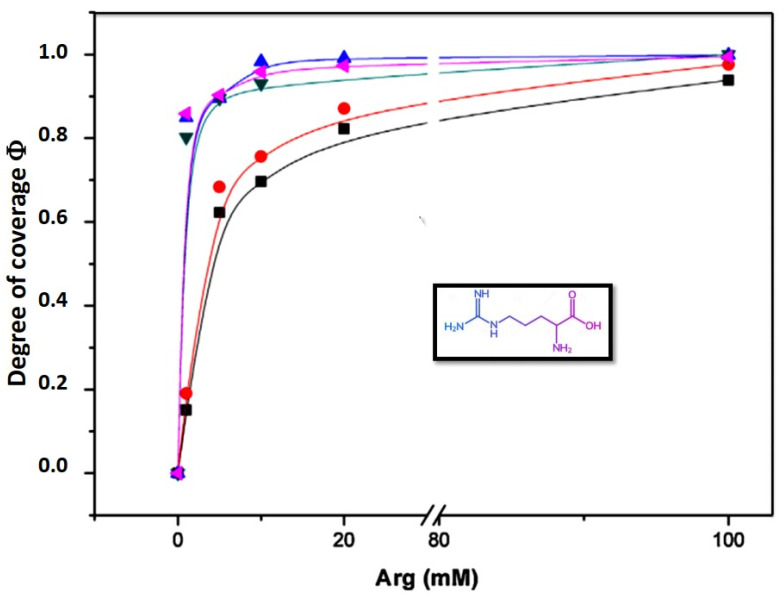
Adsorption isotherms of arginine (Arg) in lipid vesicles of DMPC, dmPE, mmPE, DMPE. Adapted from [27].

Data can be fitted plotting the degree of coverage vs. concentration (Figure 4). The degree of coverage of the liposome surface by a compound *C* can be obtained from the equilibrium between adsorbed and non-adsorbed species. The equation derived is:

(8) $$\theta =\frac{{\left(KC\right)}^{n}}{1+{\left(KC\right)}^{n}}$$

where *K* is the affinity constant of the compound, and *n* is a parameter that accounts for the heterogeneity for the adsorption process. If *n* = 1 the process obeys the Langmuir isotherm implying that the adsorption sites are energetically equal and are independent [14,26]

In turn, Equation (9) can be related with zeta potential by,

θ = ζ − ζ_o_/ζ_∞_ − ζ_o_ (9)

where ζ_o_ is the initial zeta potential value, ζ_∞_ the constant value obtained at saturation and ζ is the zeta potential for each concentration. It is clear from this relation that at the initial point θ = 0 since ζ = ζ_o_ and, at saturation, θ = 1 because ζ = ζ_∞_

From these plots important parameters in regard to the adsorption of ions and molecules to liposome surface, such as the affinity constant K, can be derived. K shows a significant difference between the Ca^2+^ adsorption to PE and PC membranes although the behavior is Langmuirian (*n* = 1) in both cases (Figure 3 Inset). When *n* (Equation (5) departs from 1, cooperative phenomena can be ascribed implying changes in membrane surface along the adsorption.

### 5.4. Aminoacid Adsorption

Following the approach and fitting described above, the behavior of zeta potential on lipid membranes composed by DMPC, DMPE, monometyl PE (mmPE) and dimethyl PE (dmPE) in the presence of Arginine is shown in Figure 4.

The values of K and *n* indicate that the affinity and the degree of cooperativity varies with the methylation of the amino group. The Langmuir isotherm (*n* = 1) only occurs in fully methylated phosphocholines (Table 1).

The adsorption of amino acids to these membranes deviates from a Langmuir type isotherm with the progressive demethylation of the phosphatidylcholine groups suggesting that the adsorption takes place in non-independent sites, probably producing surface rearrangements, changes in interfacial water and double layer organization. Zeta potential measurements provide direct evidence of amino acid orientation in a lipid interphase giving consistency to the findings suggested by molecular dynamics (MD) and surface spectroscopic studies, congruent with the proposal that the guanidine group is buried in the membrane in a water environment [28].

### 5.5. Peptide Adsorption

**Figure 5:**
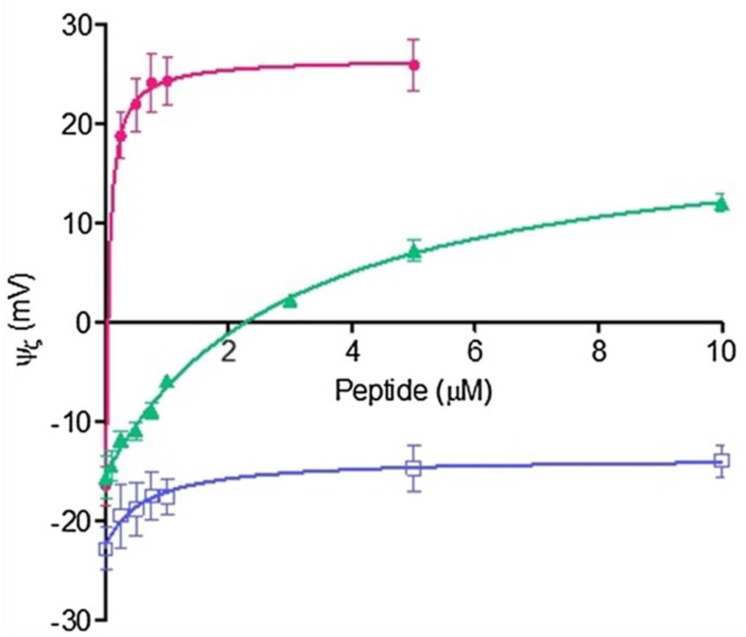
Effect of Melittin (●), Arg 7 (▲) and Lys 5 (□) on the zeta potential of DMPC liposomes. Inset: maximum values obtained at high peptide concentration. Adapted from ref. [29].

In Figure 5, the different effects of melittin, a peptide derived from bee venom, in comparison to amino acid homopeptides of seven arginines and five lysines (Arg-7 and Lys-5 respectively) on the zeta potential values are shown. Melittin produces a net displacement to positive values at very low concentrations, an effect observed at higher concentrations for Arg-5 or not observed for Lys-5 [29].

The affinity of melittin to DMPC is higher than that for the homopeptides Arg-5 and Lys-5. Although Arg and Lys are components of melittin, the effect on membrane surface seems to be a synergistic process involving a combination of amino acid motifs.

### 5.6. Zeta Potential and Enzyme Activity

Zeta potential is also a suitable methodology to follow processes at the lipid interphase. It has been found useful to follow the release of electrostatically anchored proteins from the membrane by calmodulin inhibitors lowering the potential of cationic membrane patches [30,31].

However, there are not many papers published in which zeta potential was determined during enzymatic reaction on lipid particles. Evidence that enzyme activity can affect zeta potential has been reported for phospholipase A_2_ (PLA_2_) and phospholipase C (PLC) [23,32].

**Figure 6:**
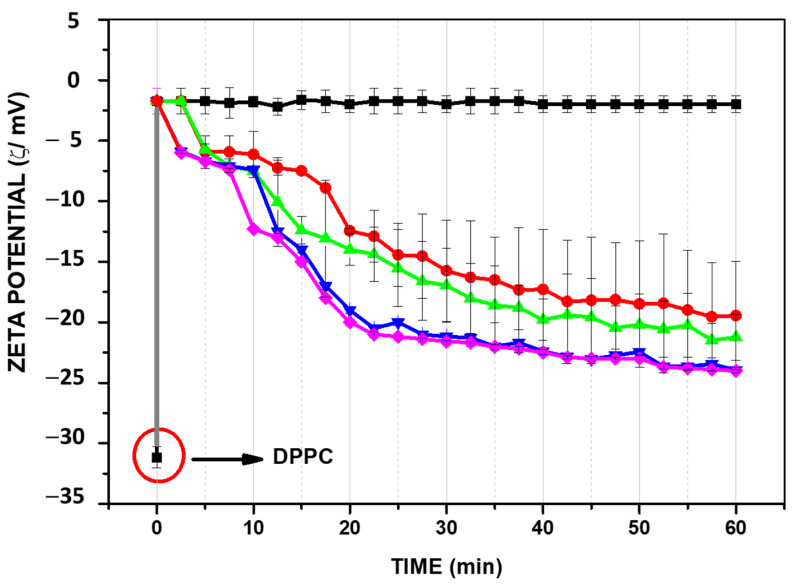
Changes of zeta potential of DPPC liposomes after the addition of PLA_2_ in the presence of Ca^+2^.

In the case of PLA_2_, the hydrolysis in the presence of Ca^+2^ produces free fatty acid and lysoPC which is manifested by a change of zeta potential towards negative values due to the accumulation of fatty acid in the membrane (Figure 6).

## 6. Dipole Potential (ψ_D_)

The origin of the dipole potential has been ascribed to the orientation of dipoles at the membrane interphase. These can be constitutive groups of the lipids (CO and PO) and water dipoles. In the scheme of Figure 1 and Figure 2, carbonyl groups of the phospholipids would be arranged in the inner plane named the hydrocarbon–water interface. This is an ideal plane running along the glycerol moieties where carbonyl groups are located [33,34].

The dipole potential has been related with the hydration forces which represents a repulsive barrier associated to the excluded volume produced by water molecules bound to lipid polar head groups [7,35,36]. Thus, a marked contribution to dipole potential is made by water dipoles bound to the polar groups by hydrogen bonds [37,38,39].

The presence of this potential originated in water arrangements is a repulsion barrier against the membrane–membrane adhesion and the adsorption of peptides and other compounds from the media [38,39]. For this reason, the magnitude of the dipole potential is important to modulate fusion processes, protein adsorption and peptide penetration. For the same reason the dehydration (i.e., decrease of dipole potential) is an important step in the mechanism of such processes.

The hydration water is responsible of the positive sign of the potential [33]. The dipole potential generates a positive charge image in the membrane interior. That is why hydrophobic anions has a permeability several orders of magnitude higher than the cationic ones [40].

### 6.1. Determination of Dipole Potential (ψ_D_)

A direct way to determine the dipole potential is in monolayers of lipids spread on the air water surface [19,21,35]. The measurement set up consists of a high impedance circuit composed by a vibrating or ionizing electrode located above the monolayer and a counter electrode of reference in the subphase.

The dipole potential is given by,

ψ_D_ = ΔV = V_lipid_ − V_solution_ (10)

where V_lipid_ is the potential obtained for the monolayer saturated in lipids and V_solution_ the potential of the surface without lipids. In this condition, a monolayer of PC shows a dipole potential of ca 400 mV. In contrast, bilayers of the same lipids show a value of 227 mV. The difference is ascribed to the use of air as a reference in monolayers [11].

### 6.2. Modification of Dipole Potential by Lipid Constituents and Physcal Chemical Variables

**Figure 7:**
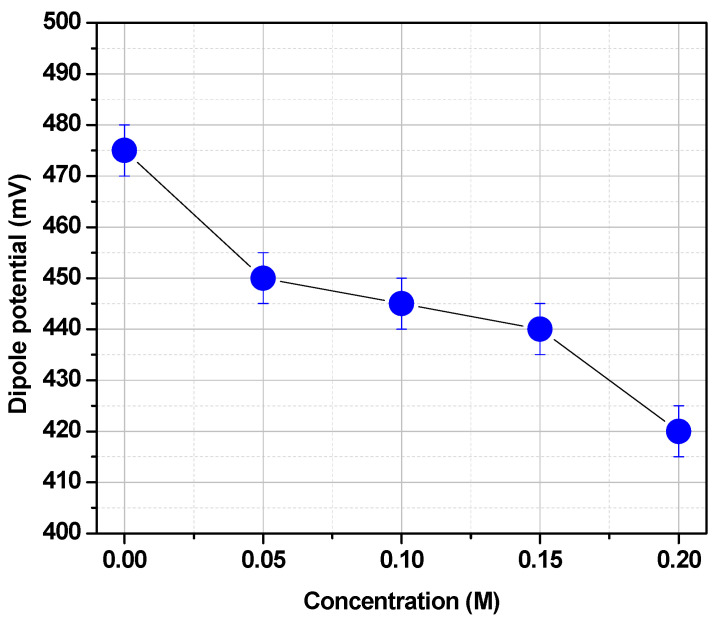
Effect of Trehalose (●) on the dipole potential of DMPC monolayers spread in an air/aqueous solution interface at 20 °C. (Adapted from ref. [37]).

The chemical structure of the lipids may change significantly the dipole potential. For instance, the lack of carbonyl groups in 16:0 di hexadecyl phosphatidylcholine (16:0 Dieter PC) decreases the DPPC dipole potential in more than 100 mV. This is clear evidence that carbonyl groups contribute to the dipole potential. However, both carbonyl and phosphate groups can form hydrogen bonds with water determining a hydration layer. This water is polarized and contributes to the dipole potential [35,37,39]. This polarized water can be displaced by poly hydroxylated compounds such as trehalose (Figure 7).

**Figure 8:**
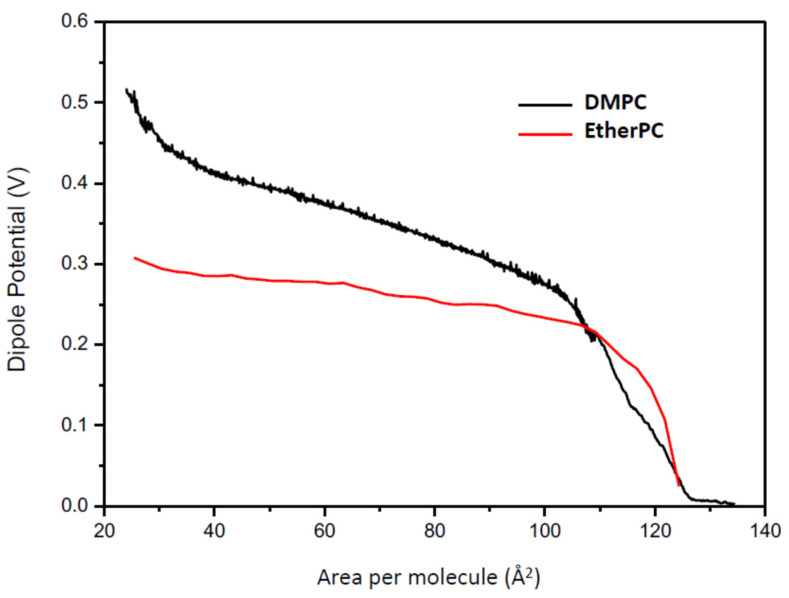
Changes in dipole potential with area for lipid with and without carbonyl groups.

The dipole potential can also be modified by the orientation of the constitutive lipids. The dipole moment is the resultant of the total dipole moment and the angle with respect to the membrane plane (Figure 8).

The carbonyl groups of glycerolipid monolayers spread on water play an important role in the formation of the interfacial hydrogen bond network which, in turn, influences the interactions of lipids with, for example, metabolites. As the frequency of the carbonyl absorption band strongly depends on the hydration state of the lipid headgroups, the carbonyl band is a sensitive reporter of changes in the headgroup environment [41]. Phase-resolved sum frequency generation spectroscopy permits to obtain information about the orientation and hydration of the carbonyl groups in lipid monolayers. There are two distinct carbonyl moieties in the lipid monolayers, oppositely oriented relative to the surface plane, that experience substantially different hydrogen-bonding environments [41,42,43].

## 7. Zeta Potential and Dipole Potential Correlation

The different contributions to the surface potential can be measured by different techniques. The results are dominated by different molecular moieties and effects: charge density, lipid carbonyl groups and the interfacial water molecules [42].

Thus, it is difficult to ascribe to only one force the energetics of the interaction of peptides and amino acids. The deviation from the Langmuir type isotherm (see Equation (5) can be indicative of the combination of different contributions to the surface potentials and hence to the adsorption and insertion processes.

This deviation is noticeable with the progressive demethylation of the phosphatidylcholine groups suggesting that the adsorption takes place in non-independent sites, probably producing surface rearrangements, changes in interfacial water and in double layer at the interphase region (Figure 2) [44].

Some models postulate that peptide insertion into lipid bilayers is mainly due to electrostatic and other specific interactions with phosphocholine head groups [29,30]. In this regard, simulation results have shown that the stabilization in the membrane of some hydrophilic groups, such as guanidinium, arginine moieties sink into the membrane phase interior by snorkeling into water pockets. Zeta potential determinations obtained from liposome electrophoretic mobilities provide direct evidence of the Arg orientation in the lipid interphase of phosphatidylcholine and phosphatidylethanolamine bilayers.

As the dipole potential generates a positive charge image in the membrane interior, the presence of a hydrophobic group in the amino acid structure produces an inversion of the zeta potential indicating that the final electrostatic surface charge is associated to the dipole potential [27,28].

A correlation of zeta and dipole potentials was done in mixtures of dimyristoylphosphatidylcholine/dipalmitoylphosphatidylcholine (DMPC/DPPC), dioleoylphosphatidylcholine/distearoylphosphatidylcholine (DOPC/DSPC), and dioleoylphosphatidylcholine/dimyristoylphosphatidylcholine (DOPC/DMPC).

Singularities in the dipole potential and the ζ potential at similar molar ratios of the mixtures in the gel state well above the experimental error were explained by similar arrangements in the surface in monolayers and bilayers [21]. The surface properties seem to be a consequence of the type and phase state of the lipids in the mixture rather than of the supramolecular organization such as monolayer or bilayer. On the other hand, they showed that both potentials are interrelated. Therefore, it is difficult to ascribe to a single force the interaction of peptides and amino acids with lipid membranes, especially those deviating for a Langmuirian adsorption.

When the dipole potential of dimyristoylphosphatidylcholine (DMPC) monolayers was decreased, either by the insertion of phloretin or by the elimination of carbonyl groups at the interphase, the surface charge potential was displaced to lower negative values which was explained by different exposure of the phosphate groups to water at low ionic strength [44]. At high ionic strength, the magnitude of the changes in the zeta potential produced by the effects on the dipole potential was found to be dependent on the type of anions present in the subphase. Differences between Cl^−^ and ClO_4_^−^ were ascribed to the adsorption of anions according to their different hydrations and polarizabilities. The influence of a low dipole potential on the anion adsorption can be ascribed to a less positive image charge at the membrane interior, resulting from an increase in the hydrocarbon core permittivity. This is congruent with the neutralization of interfacial dipoles and the area increase, as well as with the decrease in packing of the hydrocarbon groups. Phloretin did not cause changes in the dipole potential of di myristoyl phosphatidylethanolamine (DMPE) and, in consequence, no effects on the zeta potential were measured. It is concluded that changes in the inner water/hydrocarbon plane affect the electrostatic potential measured in the outer plane of the polar headgroup region.

The alignment of water molecules is linearly dependent to the electrical charges in the membrane surface at low concentration [ that saturates at physiologically relevant charge densities [42]. The saturation occurs in both the Stern layer, i.e., inside the interphase region, directly at the surface, and in the diffuse layer. This accounts for a marked reduction of the surface potential at high surface charge density via both interfacial molecular rearrangement and permeation of monovalent ions into the interface [32].

There is a great content of ambiguity for the definition of the cell limits and cell membranes. These boundaries derive in difficulties to define the limits in the membrane itself. Definitions derived from structural geometrical parameters are sometimes in conflict with thermodynamic and electrochemical criteria.

With the aid of molecular dynamics and new scattering methodologies, the limit between water and the hydrocarbon region has become diffuse. Water can extend into the first 4–5 C atoms below the carbonyl groups and from the plane of the phosphates and the different polar residues attached to it, toward the external water. Consequently, the neat differentiation of water near the groups and the bulk as described by the definition of the surface potentials is gradually diluted [1,45].

In consequence, the presence of different water organization at the membrane interphase i.e., the EDL makes the process more complex. A group of water molecules are tightly bound to the PO and CO groups (the first hydration layer). A second shell extends from this and penetrates the nonpolar region. The second shell can vary with osmotic stress and surface pressure thus modifying surface charge concentration and dipole alignment.

## 8. Conclusions

Zeta potential and dipole potential measures can be direct operational methodologies to determine the adsorption, insertion and penetration of ions, amphipathic and neutral compounds into the membrane. However, although they may give apparent affinity or binding constants care should be taken to interpret them in terms of physical meaning.

This is because both potentials are interdependent. This is to say that if a variation of zeta potential is determined it cannot be only ascribed to electrostatic forces and vice versa.

In addition, the different processes mentioned above implied interaction at different level of the membrane structure, namely, at the interphase region itself, at the inner plane of the interphase and in the interior of the hydrocarbon core. Mostly, the membrane acts as a single entity as effects can occur in all these regions at different extent and intensities.

An important derivation of the present analysis is the role of water in the electrical phenomena. It has been described how dipole potential and zeta potential are correlated which, in essence is due to water reorganization. Considering that in a cell the interphase region the membrane extends to the cell interior or overlaps with the interphase region of another supramolecular structure, as proposed in complex systems, the correlation of dipole and electrostatic forces can contribute to the propagation of perturbations between membrane and cytoplasm and vice versa [3]. Thus, this picture gives the membrane a responsive character in addition to its selective permeability barrier. It turns out that the responsive and the correlation of different types of potential along changes in water arrangements makes permeability phenomena more complex in terms of dynamics and kinetics.

## Figures and Tables

**Table 1:** Zeta potential, affinity constant and cooperativity coefficient as a function of the de methylation of phosphocholine groups.

Lipid	Zera Potential (mV)	Affinity Constant (K) M^−1^	*N*
DMPC	−13.7 ± 2.1	0.54 × 10^3^	1
dmPE	−29.9 ± 2.3	0.18 × 10^3^	1
mmPE	−39.1 ± 3.2	2.90 × 10^3^	0.9
DMPE	−45.3 ± 0.9	2.00 × 10^3^	0.74

## Data Availability

Not applicable.

## References

[1] Frias M., Disalvo E. (2020). Breakdown of classical paradigms in relation to membrane structure and functions. Biochim. Biophys. Acta Biomembr..

[2] Pinto O., Disalvo E. (2019). A new model for lipid monolayer and bilayers based on thermodynamics of irreversible processes. PLoS ONE.

[3] Bagatolli L.A., Stock R.P. (2021). Lipids, membranes, colloids and cells: A long view. Biochim. Biophys. Acta Biomembr..

[4] Viera L., Senisterra G., Disalvo E. (1996). Changes in the optical properties of liposome dispersions in relation to the interlamellar distance and solute interaction. Chem. Phys. Lipids.

[5] Galassi V.V., Wilke N. (2021). On the Coupling between Mechanical Properties and Electrostatics in Biological Membranes. Membranes.

[6] McIntosh T., Simon S., Dilger J. (1989). Location of the water-hydrocarbon interface in lipid bilayers. Water Transp. Biol. Membr..

[7] Disalvo E., De Gier J. (1983). Contribution of aqueous interphases to the permeability barrier of lipid bilayers for non-electrolytes. Chem. Phys. Lipids.

[8] Satoh K. (1995). Determination of binding constants of Ca2+, Na+, and Cl− ions to liposomal membranes of dipalmitoylphosphatidylcholine at gel phase by particle electrophoresis. Biochim. Biophys. Acta Biomembr..

[9] Liu Y., Yan E.C., Zhao X., Eisenthal K.B. (2001). Surface potential of charged liposomes determined by second harmonic generation. Langmuir.

[10] Warshaviak D.T., Muellner M.J., Chachisvilis M. (2011). Effect of membrane tension on the electric field and dipole potential of lipid bilayer membrane. Biochim. Biophys. Acta Biomembr..

[11] Clarke R.J. (2001). The dipole potential of phospholipid membranes and methods for its detection. Adv. Colloid Interface Sci..

[12] McLaughlin S., Mulrine N., Gresalfi T., Vaio G., McLaughlin A. (1981). Adsorption of divalent cations to bilayer membranes containing phosphatidylserine. J. Gen. Physiol..

[13] Bhattacharjee S. (2016). DLS and zeta potential—What they are and what they are not?. J. Control. Release.

[14] Delgado Á.V., González-Caballero F., Hunter R., Koopal L., Lyklema J. (2007). Measurement and interpretation of electrokinetic phenomena. J. Colloid Interface Sci..

[15] Langner M., Kubica K. (1999). The electrostatics of lipid surfaces. Chem. Phys. Lipids.

[16] Makino K., Yamada T., Kimura M., Oka T., Ohshima H., Kondo T. (1991). Temperature-and ionic strength-induced conformational changes in the lipid head group region of liposomes as suggested by zeta potential data. Biophys. Chem..

[17] Tatulian S.A. (1983). Effect of lipid phase transition on the binding of anions to dimyristoylphosphatidylcholine liposomes. Biochim. Biophys. Acta Biomembr..

[18] Morini M.A., Sierra M.B., Pedroni V.I., Alarcon L.M., Appignanesi G.A., Disalvo E.A. (2015). Influence of temperature, anions and size distribution on the zeta potential of DMPC, DPPC and DMPE lipid vesicles. Colloids Surf. B Biointerfaces.

[19] MacDonald R., Simon S. (1987). Lipid monolayer states and their relationships to bilayers. Proc. Natl. Acad. Sci. USA.

[20] Lavaisse L.M., Hollmann A., Nazareno M.A., Disalvo E.A. (2019). Zeta potential changes of Saccharomyces cerevisiae during fermentative and respiratory cycles. Colloids Surf. B Biointerfaces.

[21] Luzardo M.d.C., Peltzer G., Disalvo E. (1998). Surface potential of lipid interfaces formed by mixtures of phosphatidylcholine of different chain lengths. Langmuir.

[22] Kaszuba M., Corbett J., Watson F.M., Jones A. (2010). High-concentration zeta potential measurements using light-scattering techniques. Philos. Trans. R. Soc. A Math. Phys. Eng. Sci..

[23] Mohtar L., Ledesma A., Disalvo E., Frias M. (2020). Influence of carbonyl groups on the interaction of PLA2 with lipid interphases. Colloid Interface Sci. Commun..

[24] Iraolagoitia X.L.R., Martini M.F. (2010). Ca^2+^ adsorption to lipid membranes and the effect of cholesterol in their composition. Colloids Surf. B Biointerfaces.

[25] Mangelsdorf C.S., White L.R. (1998). The dynamic double layer part 2 effects of Stern-layer conduction on the high-frequency electrokinetic transport properties. J. Chem. Soc. Faraday Trans..

[26] Lyklema J., Minor M. (1998). On surface conduction and its role in electrokinetics. Colloids Surf. A Physicochem. Eng. Asp..

[27] Disalvo E., Bouchet A. (2014). Electrophoretic mobility and zeta potential of liposomes due to arginine and polyarginine adsorption. Colloids Surf. A Physicochem. Eng. Asp..

[28] Bouchet A., Lairion F., Disalvo E.A. (2010). Role of guanidinium group in the insertion of l-arginine in DMPE and DMPC lipid interphases. Biochim. Biophys. Acta.

[29] Tissera M.J.E., Disalvo E.A., Martini M.F., Cutró A.C. (2019). Filling gaps in the knowledge of melittin on lipid membranes. Colloids Surf. A Physicochem. Eng. Asp..

[30] Fan H.Y., Nazari M., Raval G., Khan Z., Patel H., Heerklotz H. (2014). Utilizing zeta potential measurements to study the effective charge, membrane partitioning, and membrane permeation of the lipopeptide surfactin. Biochim. Biophys. Acta Biomembr..

[31] Sengupta P., José M., Tebar F., Golebiewska U., Zaitseva I., Enrich C., McLaughlin S., Villalobo A. (2007). Membrane-permeable calmodulin inhibitors (eg W-7/W-13) bind to membranes, changing the electrostatic surface potential: Dual effect of W-13 on epidermal growth factor receptor activation. J. Biol. Chem..

[32] Chibowski E., Szcześ A. (2016). Zeta potential and surface charge of DPPC and DOPC liposomes in the presence of PLC enzyme. Adsorption.

[33] Zheng C., Vanderkooi G. (1992). Molecular origin of the internal dipole potential in lipid bilayers: Calculation of the electrostatic potential. Biophys. J..

[34] Simon S.A., McIntosh T.J. (1986). Depth of water penetration into lipid b: Layers. Meth. Enzym..

[35] Simon S., McIntosh T. (1989). Magnitude of the solvation pressure depends on dipole potential. Proc. Natl. Acad. Sci. USA.

[36] Parsegian V., Rand R., Rau D. (2000). Osmotic stress, crowding, preferential hydration, and binding: A comparison of perspectives. Proc. Natl. Acad. Sci. USA.

[37] Luzardo M.C., Amalfa F., Nunez A.M., Diaz S., Biondi De Lopez A.C., Disalvo E.A. (2000). Effect of trehalose and sucrose on the hydration and dipole potential of lipid bilayers. Biophys. J..

[38] Diaz S., Lairion F., Arroyo J., Biondi de Lopez A., Disalvo E. (2001). Contribution of phosphate groups to the dipole potential of dimyristoylphosphatidylcholine membranes. Langmuir.

[39] Diaz S., Amalfa F., de Lopez B., Disalvo E. (1999). Effect of water polarized at the carbonyl groups of phosphatidylcholines on the dipole potential of lipid bilayers. Langmuir.

[40] Franklin J.C., Cafiso D.S. (1993). Internal electrostatic potentials in bilayers: Measuring and controlling dipole potentials in lipid vesicles. Biophys. J..

[41] Disalvo E., Frias M. (2013). Water state and carbonyl distribution populations in confined regions of lipid bilayers observed by FTIR spectroscopy. Langmuir.

[42] Dreier L.B., Bernhard C., Gonella G., Backus E.H., Bonn M. (2018). Surface Potential of a Planar Charged Lipid–Water Interface. What Do Vibrating Plate Methods, Second Harmonic and Sum Frequency Measure?. J. Phys. Chem. Lett..

[43] Lairion F., Disalvo E.A. (2004). Effect of phloretin on the dipole potential of phosphatidylcholine, phosphatidylethanolamine, and phosphatidylglycerol monolayers. Langmuir.

[44] Lairion F., Disalvo E.A. (2009). Effect of dipole potential variations on the surface charge potential of lipid membranes. J. Phys. Chem. B.

[45] Calero C., Franzese G. (2019). Membranes with different hydration levels: The interface between bound and unbound hydration water. J. Mol. Liq..

